# Supplementary material for: Cold Adaptation in Antarctic Notothenioids: Comparative Transcriptomics Reveals Novel Insights in the Peculiar Role of Gills and Highlights Signatures of Cobalamin Deficiency
Source: Int J Mol Sci. 2021 Feb 11;22(4):1812. doi: 10.3390/ijms22041812 (PMC7918649; doi:10.3390/ijms22041812)
Supplement: Supplementary file 1 [file ijms-22-01812-s001.zip › ijms-1102425-supplementary for pub/ijms-1102425 -Supplementary_data_notes.docx]

**Supplementary Material**

**Supplementary Data Note 1: functional enrichment of gills-specific transcripts**

We performed hypergeometric tests to identify the biological processes and gene families more intimately linked to gill functions in *C. hamatus*, i.e. those connected to annotations over-represented in the gill-specific gene set. All significantly enriched annotations for Gene Ontology molecular function (MF), biological process (BP), and cellular component (CC) categories, as well as for Pfam conserved domains, are listed in Table S1. As described below, the vast majority of these annotations find a justification in physiological functions and morphological features common to the gills of all teleosts, independently from their life environment, range of distribution, and adaptation to diverse temperature ranges.

For example, genes encoding structural components of gills fibrils, like several keratins (in particular a few highly expressed keratin-13 homologs, and keratin-8, Figure 2), emerge due to their localized expression in the gills. Cytoskeletal keratins, as the main constituents of intermediate filaments, enable high deformability and stretching of cells, in particular in epithelial tissues. Keratins have been previously shown to be major components of the epithelial layer of fish gills, both by chemical staining [1,2] and gene expression studies [3]. Intermediate filaments (Table S1), together with a series of extracellular cellular matrix proteins mediating cell–cell contact, may play a fundamental role in establishing and maintaining the fibrillary organization of the gills tissue, establishing a mucosal barrier, which also represents one of the major interfaces with the external environment.

The gills epithelium is known to play a key role in salt and water balance in marine fishes, as it is “leaky” to facilitate the secretion of Na^+^ ions. The high paracellular permeability properties of gills are enabled by structural components of the tight junction complex [4], which are highly represented in the *C. hamatus* transcriptome. Several different genes pertaining to the claudin superfamily (Table S1) and part of the tight junction complex were gills-specific, consistently with their prominent role in the establishment and maintenance of the barrier properties of the fish gill epithelium [5].

The finding that many highly expressed gills-specific transcripts are involved in immune response was not surprising. Indeed, this is linked to the presence of GIALT, which contains a high fraction of B [6] and T cells [7,8], macrophages and granulocytes [9,10]. The presence of many highly expressed “gill-specific” genes related to immune functions (Figure 2) is further explained by the fact that none of the other tissues currently available for a comparative gene expression analysis in *C. hamatus* (skeletal muscle, heart, liver, and head kidney), is involved in mucosal immunity. Only the head kidney produces a relevant amount of transcripts related to immunity in Antarctic fishes, even though these are mostly related to the hematopoietic function of that tissue [11].

Among the most highly expressed transcripts involved in immune processes (Figure 2), the presence of two contigs related to Class I histocompatibility antigen is remarkable. These heterodimeric membrane-associated proteins bind and expose peptides generated from the proteasomal degradation of cytosolic proteins, but they can also potentially expose fragments derived from the degradation of exogenous proteins from invading microbes through cross-presentation, an immune pathway which covers a fundamental importance in some fish lineages [12].

Several GTPases linked to immune signal transduction and to the modulation of cellular and systemic immune response are also found in the gill-specific gene subset (Figure 2). Among these, the presence of many GTPases pertaining to the AIG1 family is worth of a note. GIMAP4 and GIMAP5 are considered as a markers of T- and B-lymphocytes in peripheral blood, as they contribute to the homeostasis and survival of these cells [13], and interferon-inducible GTPase 5 belongs to a large gene family involved in the regulation of host defense systems [14].

The expression of trypsin-like proteases was also found to be enhanced in gills compared to other tissues, with TPSAB1 being the most highly expressed gene of this family (Figure 2). This is explained by the important role such enzymes cover as accessory factors in protection from bleeding [15]. Indeed, the gill tissue is highly vascularized and subjected to high blood pressure, in particular in Antarctic notothenioids. The direct contact of gills with the external environment potentially exposes them to injury, which could be fatal in absence of efficient mechanisms for the activation of thrombocytes.

Another over-represented gene family encodes proteins sharing structural similarity with hemolytic pore-forming toxins produced by hydrozoans (Table S1), whose function is presently unknown. Indeed, fish actinoporins, unlike their cnidarian counterparts, lack permeabilizing properties [16].

Intriguingly, two out of the twenty highly expressed gill-specific protein-coding transcripts are annotated as carbonic anhydrases (CA) (Figure 2). CAs and their specific roles in Cryonotothenioidea gills are discussed in detail in Section 2.5.

| **Type** | **Code** | **Annotation** | **p-value** |
| --- | --- | --- | --- |
| PFAM | PF04548 | AIG1 family | 1E-16 |
| CC | 42612 | MHC class I protein complex | 9E-15 |
| PFAM | PF00129 | Class I Histocompatibility antigen. domains alpha 1 and 2 | 2E-13 |
| MF | 5198 | structural molecule activity | 3E-13 |
| BP | 1916 | positive regulation of T cell mediated cytotoxicity | 7E-12 |
| MF | 42605 | Antigen processing and presentation of peptide antigen via MHC class I | 8E-12 |
| PFAM | PF00038 | Intermediate filament protein | 6E-11 |
| BP | 2474 | antigen processing and presentation of peptide antigen via MHC class I | 2E-09 |
| BP | 46931 | pore complex assembly | 3E-09 |
| MF | 5525 | GTP binding | 5E-08 |
| BP | 52331 | hemolysis in other organism involved in symbiotic interaction | 1E-07 |
| PFAM | PF00089 | Trypsin | 1E-07 |
| PFAM | PF06369 | Sea anemone cytotoxic protein | 1E-07 |
| CC | 44218 | other organism cell membrane | 2E-07 |
| CC | 42151 | nematocyst | 2E-07 |
| CC | 5882 | intermediate filament | 4E-07 |
| BP | 44179 | hemolysis in other organism | 2E-06 |
| CC | 30057 | desmosome | 3E-06 |
| MF | 15267 | channel activity | 4E-06 |
| BP | 61436 | establishment of skin barrier | 7E-06 |
| PFAM | PF13365 | Trypsin-like peptidase domain | 1E-05 |
| MF | 4252 | serine-type endopeptidase activity | 2E-05 |
| CC | 70062 | extracellular vesicular exosome | 2E-05 |
| PFAM | PF00822 | PMP-22/EMP/MP20/Claudin family | 3E-05 |
| PFAM | PF00656 | Caspase domain | 4E-05 |
| PFAM | PF01926 | 50S ribosome-binding GTPase | 5E-05 |
| CC | 5576 | extracellular region | 5E-05 |
| CC | 30658 | transport vesicle membrane | 0.0001 |
| BP | 15031 | protein transport | 0.0003 |
| BP | 51603 | proteolysis involved in cellular protein catabolic process | 0.0003 |
| BP | 6915 | apoptotic process | 0.0004 |
| CC | 5811 | lipid particle | 0.0005 |
| PFAM | PF03372 | Endonuclease/Exonuclease/phosphatase family | 0.0007 |
| CC | 5793 | endoplasmic reticulum-Golgi intermediate compartment | 0.0007 |
| BP | 6955 | immune response | 0.0008 |
| PFAM | PF13765 | SPRY-associated domain | 0.0009 |
| MF | 5509 | calcium ion binding | 0.0018 |
| PFAM | PF00025 | ADP-ribosylation factor family | 0.0026 |
| MF | 4197 | cysteine-type endopeptidase activity | 0.0027 |
| CC | 32580 | Golgi cisterna membrane | 0.0029 |
| CC | 5923 | tight junction | 0.0031 |
| CC | 30670 | phagocytic vesicle membrane | 0.0034 |
| PFAM | PF13405 | EF-hand domain | 0.0051 |
| CC | 5764 | lysosome | 0.0057 |
| PFAM | PF00036 | EF hand | 0.0062 |
| CC | 16324 | apical plasma membrane | 0.0067 |
| PFAM | PF08477 | Ras of Complex. Roc. domain of DAPkinase | 0.0082 |
| MF | 3924 | GTPase activity | 0.02 |
| MF | 42802 | identical protein binding | 0.02 |
| PFAM | PF00071 | Ras family | 0.02 |

**Table S1**: List of the significantly over-represented annotations in the set of gill-specific genes. BP: Gene Ontology Biological Process; MF: Gene Ontology Molecular Function; CC: Gene Ontology Cellular Component; PFAM: PFAM domains.

**Supplementary Data Note 2: validation of gene expression profiles through qRT-PCR**

Six transcripts, marked as gills-specific based on RNA-seq data and on the criteria defined in Section 3.2 (see main text), were selected for validation through qRT-PCR on four additional adult individuals, taking into account five different tissues (Table S2).

| **Target gene** | **Acronym** | **Forward primer sequence** | **Reverse primer sequence** |
| --- | --- | --- | --- |
| Elongation factor 1 alpha* | EF1A | TCTCAAGCTACCCTCCCCTC | TTCCGGAAGCATCCTTGGTC |
| 40S ribosomal protein S7* | RPS7 | CTTGGAGGACCTGGTGTTCC | TCTGCTGAGCTTTGTCCAGG |
| Carbonic anhydrase 1a | CA1a | CATATGACGGCTCCCTGACC | CTGCTCACAGCTGACACTGA |
| Carbonic anhydrase 4a | CA4a | GCAATCTGTCTCCACCCAGT | CTAACGCCTCCCAGAACAAG |
| Transcobalamin-like protein | TCNL | ACCGACACTCCCAACAAGAC | GGATCCTCGGTGTAGGTGAA |
| Keratin, type II cytoskeletal 8 | KRT8 | ACATCGAAATCGCCACCTAC | GGACACACCACCAGAGGACT |
| Tryptase-like protease | TPSAB1 | GTCAGGGAGACTCAGGTGGA | TCCAGGAAAATATGGCAAGG |
| Putative antifreeze C-type lectin | AF-CTL | TGCTGAGAGAGATTGCCTTGG | GATCTTGGCCTGTTGCTGTTCC |

**Table S2**: List of primers used for the validation of tissue specificity of six target genes identified as gills-specific by RNA-seq data analysis. The complete name of target genes, as well as the acronym used to designate them in the figures are displayed. Primer sequences are reported in a 5’- 3’ direction. * indicates the housekeeping genes used for normalization purposes.

Although some degree of inter-individual variability in gene expression levels was evident, the profiles obtained with this methodology were coherent with those observed from whole-transcriptome sequencing, confirming that the sequencing data obtained in this study provided a trustworthy representation of the *C. hamatus* gill transcriptomic landscape (Figure S1). However, we cannot exclude that the marked tissue-specificity of some of the genes whose pattern of expression could not be validated with this approach are linked with factors affecting the physiology of the single individual subjected to RNA-sequencing, such as age, sex, feeding status, and presence of undocumented pathological conditions.

In detail, all the six selected genes displayed (i) extremely high expression values in gills, several folds higher than the selected housekeeping genes; (ii) a limited expression in other tissues, usually more than 10 folds lower than gills. While these observations are fully consistent with the data collected from RNA-sequencing, some discrepancies between transcriptome data and RT-PCR were evident. The most notable among these was certainly Transcobalamin-like protein (TCNL), which was ranked as the most highly expressed gene in gills based on transcriptome data (Figure 2), but displayed an expression level significantly lower than other target genes in the RT-PCR experiment (e.g. CA1A, CA4A, TPSAB1, and KRT8) (Figure S1). Such differences may have different explanations, either residing in inter-individual differences in gene expression levels, linked to the sex and age, physiological conditions of the specimens, or to the intrinsic technical differences between the two methods. In spite of the extremely stable conditions of Antarctic waters, differences in environmental parameters might have also played a role in determining these discrepancies (the individual used for RNA-sequencing was collected in 2009, while those used for qRT-PCR in 2017). More in general, while the results of RNA-seq and qRT-PCR methods usually display a good correlation, they are characterized by different dynamic ranges, and while qRT-PCR potentially permits to achieve higher accuracy, the quantification is limited to a single isoform, while RNA-seq cumulatively accounts for gene expression of all the alternatively spliced isoforms of a gene [17].


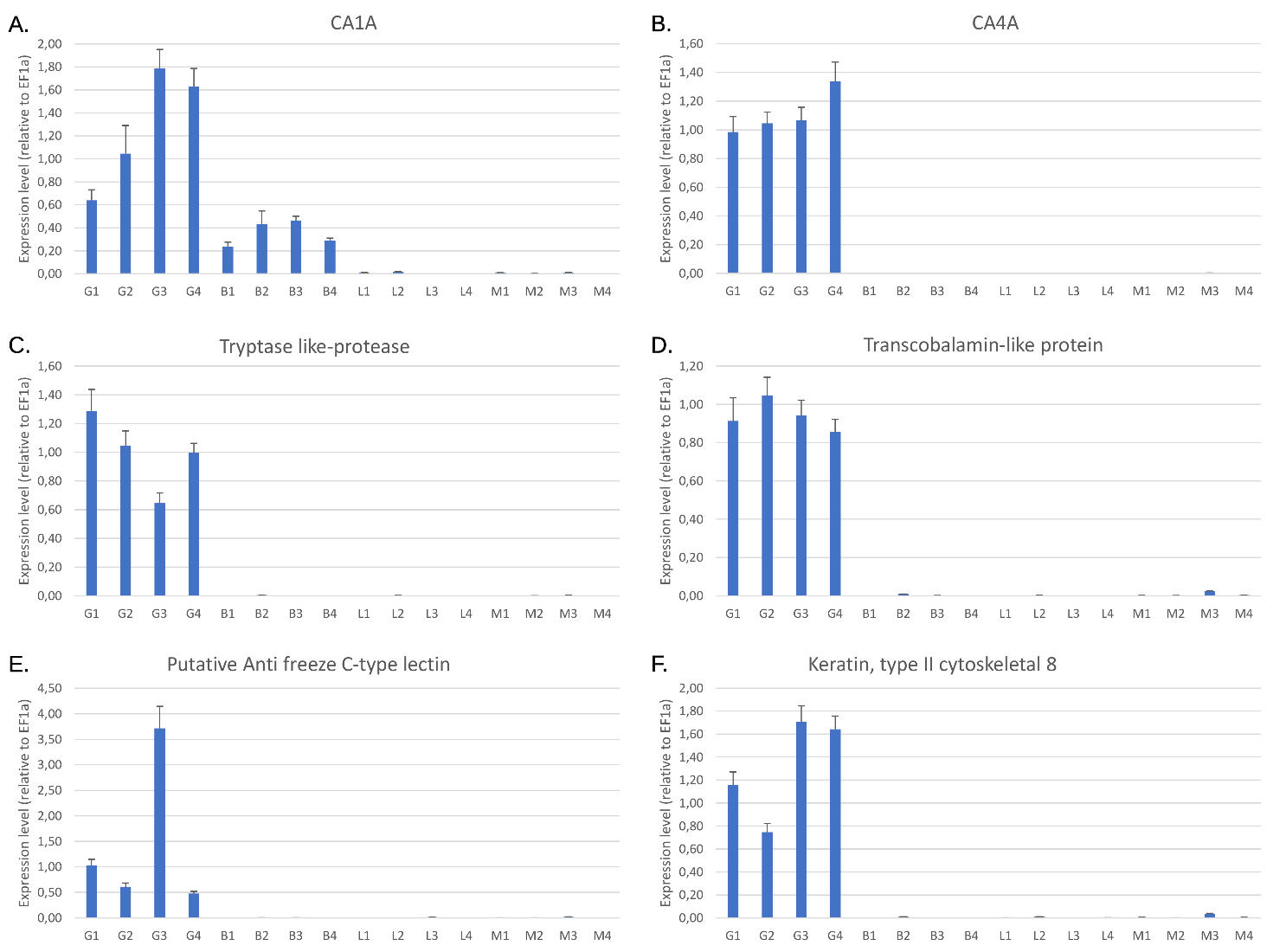


**Figure S1**: Expression levels of the six gills-specific transcripts selected for validation (Table S2), calculated in four biological replicates. Expression values are relative to the two reference stable housekeeping genes, EF1A and RPS7. Bars indicate the mean plus standard deviation of three technical replicates. G: gills; B: brain; L: liver; M: skeletal muscle.

**Supplementary Data Note 3: In silico confirmation of gill-specificity in *Trematomus bernacchii***

The availability of RNA-seq data from 19 different adult tissues allowed us to assess whether the genes identified as strongly gills-specific maintained their remarkable tissue specificity also in *T. bernacchii* (see Section 3.2 for methodological details). As exemplified below for six target genes (Figure S2), most of the icefish gill-specific transcripts displayed a strong specificity also in the red-blooded emerald rockcod, with poor or even no detectable expression in the other tissues.

In detail, the carbonic anhydrase CA1A (panel A) was expressed in all tissues, but at values from ~5 (optic tectum) to more than 100 folds (eye) lower than gills. A second carbonic anhydrase, CA4A, (panel B) was not expressed at all in most tissues, only reaching significant transcriptional levels in gonads (~5 folds lower than gills). Similarly, the transcobalamin-like protein TCNL (panel C) was selectively expressed in gills and was only transcribed at nearly-negligible levels in other tissues. The tryptase-like gene TPSAB1 (panel D) was only transcriptionally active in two tissues, i.e. gills and, at levels lower by ~95%, in liver. The keratin gene KRT8 (panel E) was mostly active in gills, but also reached biologically relevant levels of expression (lower by 80–95%) in several other tissues. The only target gene showing a somewhat broader range of expression was the putative antifreeze C-type lectin AF-CTL (panel F); despite being highly expressed in *T. bernacchi* gills, this gene was also expressed at similar or slightly higher levels in spleen, head kidney, and heart.


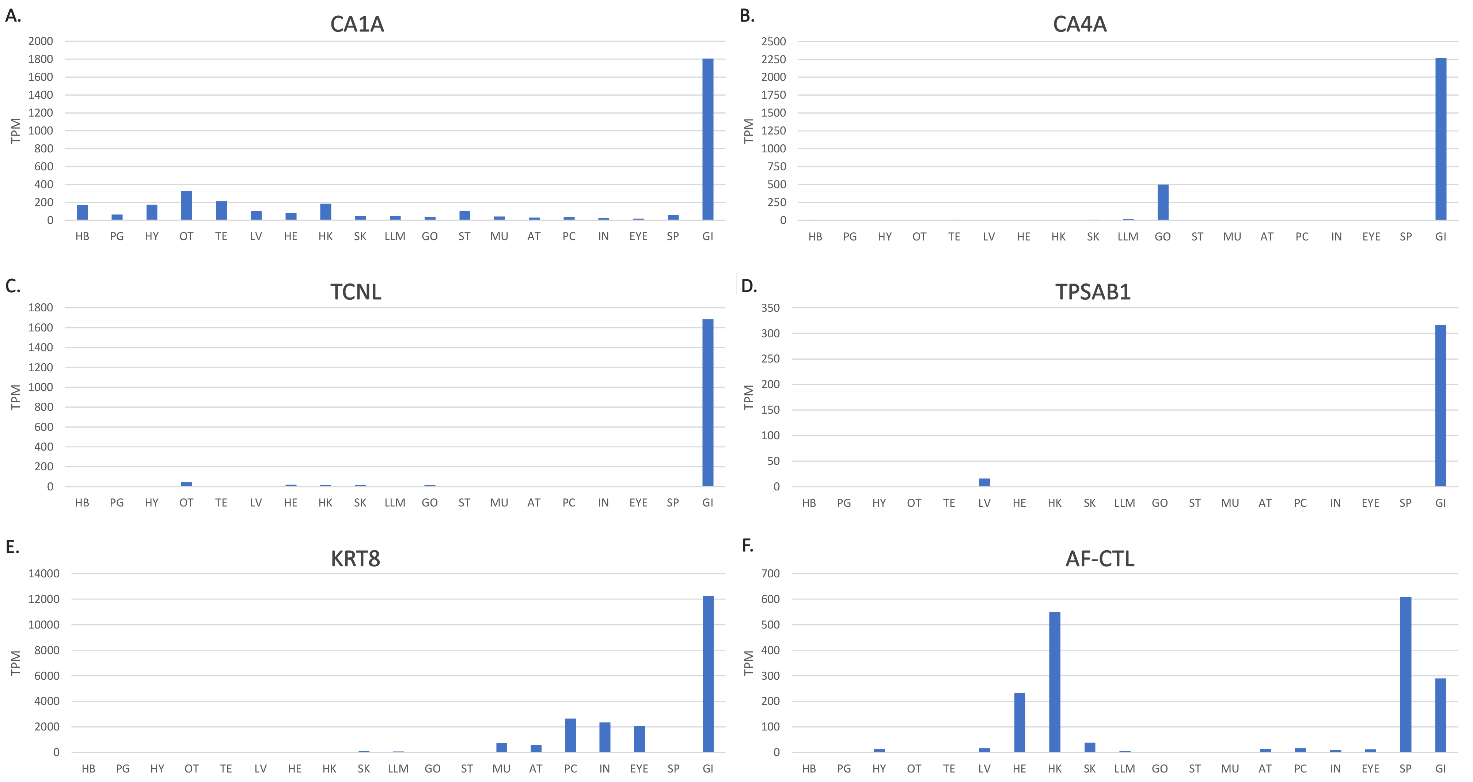


**Figure S2**: HB: hind brain; PG: pituitary gland; HY: hypothalamus; OT: optic tectum; TE: telencephalon; LV: liver; HE: heart; HK: head kidney; SK: skin; LLM: lateral line muscle; GO: gonad; ST: stomach; MU: muscle; AT: adipose tissue; PC: pyloric caecum; IN: intestine; EYE: eye; SP: spleen; GI: gills.

**Supplementary Data Note 4: comparison between the gills gene expression profiles of Cryonotothenioidea and non-Antarctic Eupercaria**

The comparative gene expression analysis carried out in this study targeted the species included in Table S3 below.

| **Species name** | **Bioproject accession** | **Taxonomical classification*** | **Environment/Climate/Range**** |
| --- | --- | --- | --- |
| *Chionodraco hamatus* | PRJNA343733 | Perciformes, Channichthyidae | Antarctic; marine/demersal |
| *Dicentrarchus labrax* | PRJNA435264 | Perciformes, Moronidae | Subtropical; marine/brackish/demersal |
| *Dissostichus mawsoni* | PRJNA401363 | Perciformes, Nototheniidae | Antarctic; marine/pelagic/oceanic |
| *Eleginops maclovinus* | PRJNA401363 | Perciformes, Eleginopsidae | Sub-Antarctic; marine/benthopelagic |
| *Gasterosteus aculeatus* | PRJNA163141 | Gasterosteiformes, Gasterosteidae | Temperate; marine/freshwater/brackish/benthopelagic |
| *Larimichthys polyactis* | PRJNA301643 | Perciformes, Sciaenidae | Subtropical; marine/benthopelagic |
| *Lateolabrax japonicus* | PRJNA302706 | Perciformes; Lateolabracidae | Subtropical; freshwater/brackish/reef-associated |
| *Lutjanus guttatus* | PRJNA395228 | Perciformes; Lutjanidae | Tropical; marine/reef-associated |
| *Pagothenia borchgrevinki* | PRJNA294774 | Perciformes, Nototheniidae | Antarctic; marine/pelagic/oceanic |
| *Parachaenichthys charcoti* | PRJEB26835 | Perciformes; Bathydraconidae | Antarctic; marine/demersal |
| *Perca fluviatilis* | PRJNA256973 | Perciformes, Percidae | Temperate; freshwater/brackish/demersal |
| *Takifugu rubripes* | PRJNA222262/PRJNA224763 | Tetraodontiformes, Tetraodontidae | Temperate; marine/brackish/demersal |
| *Trematomus bernacchii* | PRJNA289753 | Perciformes, Nototheniidae | Antarctic; marine/demersal |
| *Trematomus newnesi* | PRJNA294787 | Perciformes, Nototheniidae | Antarctic; marine/demersal |

**Table S3**: Summary of the species and sequence data taken into account in this study. *Order and family classifications are shown. **based on FishBase data, <http://www.fishbase.org>.

Overall, the gills gene expression profiles, evaluated based on the set of BUSCOs shared by all Actinopterygii, resulted in an evident distinction between Antarctic and non-Antarctic species, as displayed by the principal component analysis shown in **Figure S3**. Cryonotothenioidea were clearly separated from non-Antarctic Eupercaria based on PC1 (which explained 26% of variance), regardless of the presence or absence of erythrocytes. The sub-Antarctic notothenioid *E. maclovinus* was placed among the non-Antarctic Eupercaria. PC2 (explaining 11.4% of variance) did not correlate with an adaptation to the Antarctic environment.

A complete overview of the genes differentially expressed in the comparison between Cryonotothenioidea and non-Antarctic Eupercaria is provided in Table S4 (see Excel file attached).


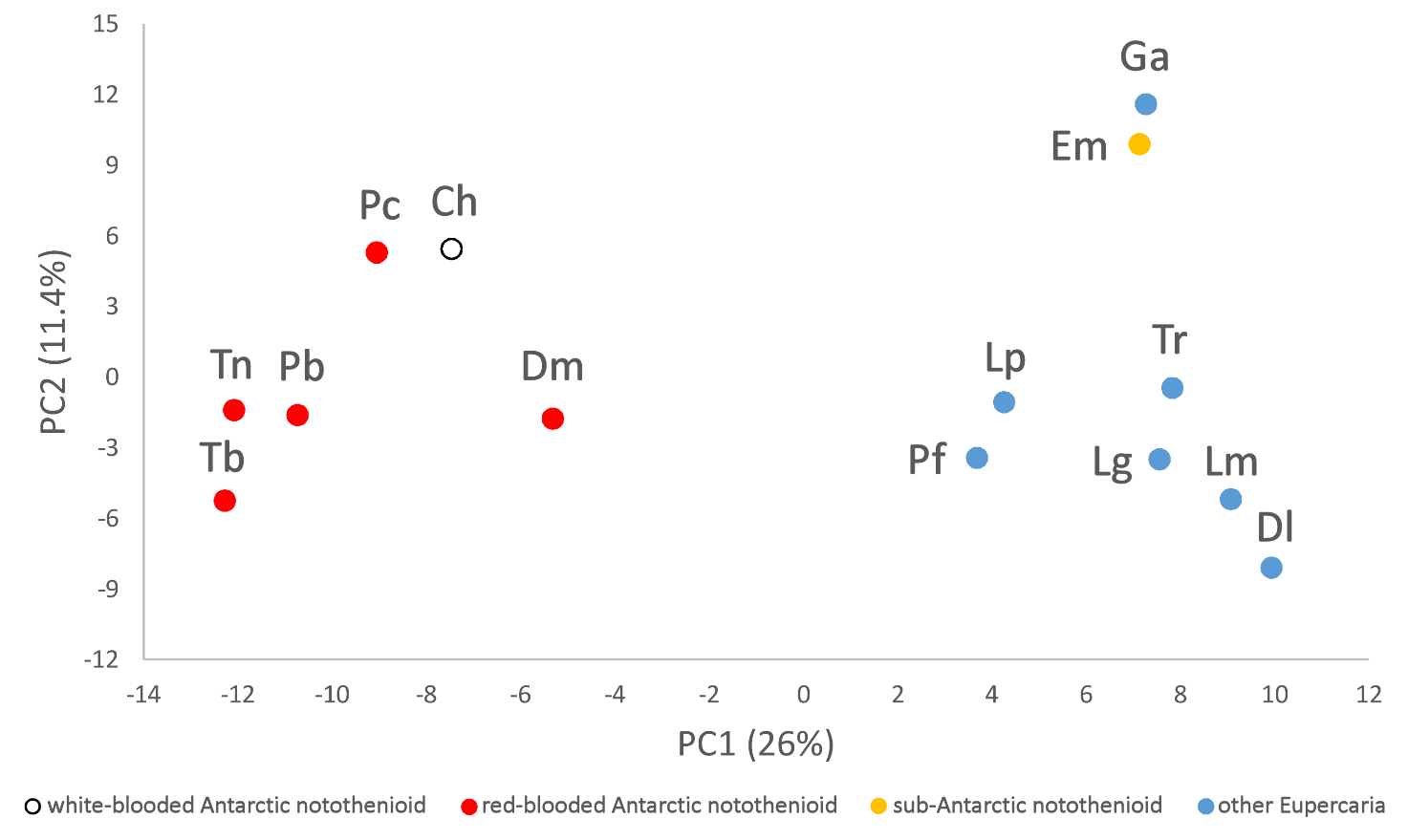


**Figure S3**: Principal component analysis of gills gene expression profiles in the 14 teleost species subject of this study. PCA was constructed based on the TPM expression levels of the 300 most highly expressed BUSCOs in the tissue. Ch: *C. hamatus*; Dl: *D. labrax*; Dm: *D. mawsoni*; Em: *E. maclovinus*; Ga: *G. aculeatus*; Lg, *L. guttatus*; Lm: *L. maculatus*; Lp: *L. polyactis*; Pb: *P. borchgrevinki*; Pc: *P. charcoti*; Pf: *P. fluviatilis*; Tb: *T. bernacchii*; Tn: *T. newnesi*; Tr: *T. rubripes*.

**Table S4**: See Excel file attached. List of the 120 differentially expressed genes resulting from the comparison between six Cryonotothenioidea and eight non-Antarctic Eupercaria species. All differentially expressed genes displayed an FDR-corrected p-value lower than 0.05 and a consistency score (see main text, section 3.3) >= 36. The table reports the gene expression levels for each species measured as TPMs.

**Supplementary Data Note 5: Characterization of the transcobalamin-like gene TCNL**

We explore the relatedness of the *Chionodraco hamatus* transcobalamin-like sequence, highly expressed in gills and one of the most relevant candidates for cold adaptation in Cryonotothenioidea, with human and fish transcobalamins. The TCN and TCNL sequences from the fish species included in this study were extracted from the transcriptome assemblies presented in the present work. The accession IDs of the human sequences are P20061, P20061, and P27352, respectively. All sequences were aligned with MUSCLE [18], and the multiple sequence alignment was refined with Gblocks [19]. The analysis was run with MrBayes v.3.2.7a [20], running two parallel MCMC chains with 500,000 generations each. Run convergence was checked by the reaching of an ESS >= 200 for all estimated parameters with Tracer v.1.7.1 [21]. The best-fitting model of molecular evolution was found to be WAG+G with modeltest-NG v.0.1.5 [22].

Cryonotothenioidea, like all other Eupercaria, display the presence of a single ortholog to human TCN1, TCN2, and GIF, plus a single TCNL gene (Figure S4). The proteins encoded by the TCN homologs shares the same domain architecture of human TCN1, TCN2, and GIF, which includes an N-terminal cobalamin-binding domain (PF01122) accompanied with a C-terminal DUF4430 domain (PF14478). Such domains are also, more simply, defined as α and β domains [23].

On the other hand, no TCNL orthologs could be found in human, and all fish TCNLs were placed in a well-supported monophyletic clade. Unlike canonical transcobalamins, TCNLs only show the C-terminal β domain, but entirely lack the α domain, which is replaced by a low complexity region (Figure S4).

Considering that the 5’ end of the icefish TCNL transcript was incomplete, likely due to technical issues during incorrectly de novo assembling of such an highly repetitive region, we reconstructed the full-length sequence by re-annotating the gene in the genome of the congeneric species *C. myersi* [24]. This was done by using the *large-gapped mapping* and *transcript discovery* tools included in the CLC Genomics Workbench 20 (Qiagen, Hilden, Germany), and by aligning *C. hamatus* gill RNA-seq data to the genomic scaffold containing the gene (i.e. scf7180000582274, identified by BLASTn using the partial *C. hamatus* sequence as a search query).

The encoding gene was found to be 2.5 Kb long, including five exons, four of which (exons 2-5) covered the complete CDS. The length of the encoded protein was 329 amino acids, with an N-terminal signal for secretion, followed by a relatively long repetitive, low complexity region, and by the β (DUF4430) domain. In human, both domains are capable of independently binding cobalamin, whose efficient transportation does however occur by encapsulation, following the interaction with both the α and β domains [23].


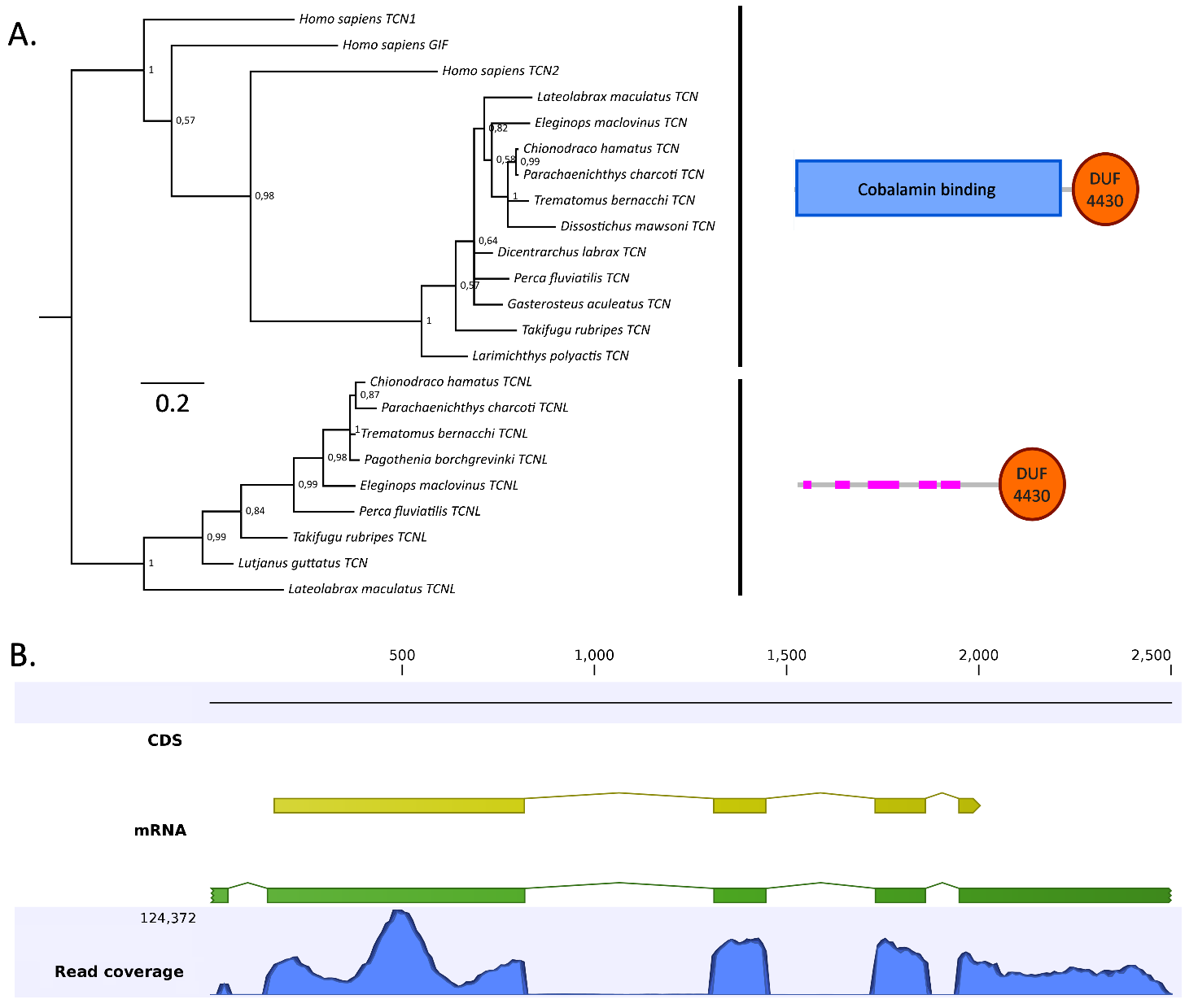


**Figure S4**: Panel A: Bayesian phylogeny of notothenioid transcobalamin (TCN) and transcobalamin-like (TCNL) proteins. A schematic view of domain organization is also reported. Panel B: Schematic structure of the *C. myersi* TCNL gene. Exon/intron organization is shown, along with the location of the CDS and the mapping profile of the RNA-seq reads obtained from the gills of *C. hamatus*.

**References**

1. Haugarvoll, E.; Bjerkås, I.; Nowak, B.F.; Hordvik, I.; Koppang, E.O. Identification and Characterization of a Novel Intraepithelial Lymphoid Tissue in the Gills of Atlantic Salmon. *J. Anat.* **2008**, *213*, 202–209, doi:10.1111/j.1469-7580.2008.00943.x.

2. Misty Paig-Tran, E. w.; Summers, A. p. Comparison of the Structure and Composition of the Branchial Filters in Suspension Feeding Elasmobranchs. *Anat. Rec.* **2014**, *297*, 701–715, doi:10.1002/ar.22850.

3. Infante, C.; Manchado, M.; Asensio, E.; Cañavate, J.P. Molecular Characterization, Gene Expression and Dependence on Thyroid Hormones of Two Type I Keratin Genes (SseKer1 and SseKer2) in the Flatfish Senegalese Sole (Solea SenegalensisKaup). *BMC Dev. Biol.* **2007**, *7*, 118, doi:10.1186/1471-213X-7-118.

4. Chasiotis, H.; Kolosov, D.; Bui, P.; Kelly, S.P. Tight Junctions, Tight Junction Proteins and Paracellular Permeability across the Gill Epithelium of Fishes: A Review. *Respir. Physiol. Neurobiol.* **2012**, *184*, 269–281, doi:10.1016/j.resp.2012.05.020.

5. Kolosov, D.; Donini, A.; Kelly, S.P. The Role Of Several Prominent Claudin Tight Junction Proteins In Teleost Fish Gill Epithelium Paracellular Permeability. *FASEB J.* **2016**, *30*, 1223.26-1223.26.

6. Salinas, I.; Zhang, Y.-A.; Sunyer, J.O. Mucosal Immunoglobulins and B Cells of Teleost Fish. *Dev. Comp. Immunol.* **2011**, *35*, 1346–1365, doi:10.1016/j.dci.2011.11.009.

7. Scapigliati, G.; Romano, N.; Abelli, L. Monoclonal Antibodies in Fish Immunology: Identification, Ontogeny and Activity of T- and B-Lymphocytes. *Aquaculture* **1999**, *172*, 3–28, doi:10.1016/S0044-8486(98)00440-2.

8. Nuñez Ortiz, N.; Gerdol, M.; Stocchi, V.; Marozzi, C.; Randelli, E.; Bernini, C.; Buonocore, F.; Picchietti, S.; Papeschi, C.; Sood, N.; et al. T Cell Transcripts and T Cell Activities in the Gills of the Teleost Fish Sea Bass (Dicentrarchus Labrax). *Dev. Comp. Immunol.* **2014**, *47*, 309–318, doi:10.1016/j.dci.2014.07.015.

9. Barnett, R.R.; Akindele, T.; Orte, C.; Shephard, K.L. Eosinophilic Granulocytes in the Epidermis of Oreochromis Mossambicus Gill Filaments Studied in Situ. *J. Fish Biol.* **1996**, *49*, 148–156, doi:10.1111/j.1095-8649.1996.tb00011.x.

10. Mulero, I.; Pilar Sepulcre, M.; Roca, F.J.; Meseguer, J.; García-Ayala, A.; Mulero, V. Characterization of Macrophages from the Bony Fish Gilthead Seabream Using an Antibody against the Macrophage Colony-Stimulating Factor Receptor. *Dev. Comp. Immunol.* **2008**, *32*, 1151–1159, doi:10.1016/j.dci.2008.03.005.

11. Gerdol, M.; Buonocore, F.; Scapigliati, G.; Pallavicini, A. Analysis and Characterization of the Head Kidney Transcriptome from the Antarctic Fish Trematomus Bernacchii (Teleostea, Notothenioidea): A Source for Immune Relevant Genes. *Mar. Genomics* **2015**, *20*, 13–15, doi:10.1016/j.margen.2014.12.005.

12. Malmstrøm, M.; Jentoft, S.; Gregers, T.F.; Jakobsen, K.S. Unraveling the Evolution of the Atlantic Cod’s (Gadus Morhua L.) Alternative Immune Strategy. *PLoS ONE* **2013**, *8*, e74004, doi:10.1371/journal.pone.0074004.

13. Schwefel, D.; Arasu, B.S.; Marino, S.F.; Lamprecht, B.; Köchert, K.; Rosenbaum, E.; Eichhorst, J.; Wiesner, B.; Behlke, J.; Rocks, O.; et al. Structural Insights into the Mechanism of GTPase Activation in the GIMAP Family. *Struct. Lond. Engl. 1993* **2013**, *21*, 550–559, doi:10.1016/j.str.2013.01.014.

14. Kim, B.-H.; Shenoy, A.R.; Kumar, P.; Bradfield, C.J.; MacMicking, J.D. IFN-Inducible GTPases in Host Defense. *Cell Host Microbe* **2012**, *12*, 432–444, doi:10.1016/j.chom.2012.09.007.

15. Kim, S.; Carrillo, M.; Kulkarni, V.; Jagadeeswaran, P. Evolution of Primary Hemostasis in Early Vertebrates. *PloS One* **2009**, *4*, e8403, doi:10.1371/journal.pone.0008403.

16. Gutiérrez-Aguirre, I.; Trontelj, P.; Macek, P.; Lakey, J.H.; Anderluh, G. Membrane Binding of Zebrafish Actinoporin-like Protein: AF Domains, a Novel Superfamily of Cell Membrane Binding Domains. *Biochem. J.* **2006**, *398*, 381–392, doi:10.1042/BJ20060206.

17. SEQC/MAQC-III Consortium A Comprehensive Assessment of RNA-Seq Accuracy, Reproducibility and Information Content by the Sequencing Quality Control Consortium. *Nat. Biotechnol.* **2014**, *32*, 903–914, doi:10.1038/nbt.2957.

18. Edgar, R.C. MUSCLE: Multiple Sequence Alignment with High Accuracy and High Throughput. *Nucleic Acids Res.* **2004**, *32*, 1792–1797, doi:10.1093/nar/gkh340.

19. Talavera, G.; Castresana, J. Improvement of Phylogenies after Removing Divergent and Ambiguously Aligned Blocks from Protein Sequence Alignments. *Syst. Biol.* **2007**, *56*, 564–577, doi:10.1080/10635150701472164.

20. Huelsenbeck, J.P.; Ronquist, F. MRBAYES: Bayesian Inference of Phylogenetic Trees. *Bioinforma. Oxf. Engl.* **2001**, *17*, 754–755.

21. Rambaut, A.; Drummond, A.J.; Xie, D.; Baele, G.; Suchard, M.A. Posterior Summarization in Bayesian Phylogenetics Using Tracer 1.7. *Syst. Biol.* **2018**, *67*, 901–904, doi:10.1093/sysbio/syy032.

22. Darriba, D.; Posada, D.; Kozlov, A.M.; Stamatakis, A.; Morel, B.; Flouri, T. ModelTest-NG: A New and Scalable Tool for the Selection of DNA and Protein Evolutionary Models. *Mol. Biol. Evol.* **2020**, *37*, 291–294, doi:10.1093/molbev/msz189.

23. Wuerges, J.; Garau, G.; Geremia, S.; Fedosov, S.N.; Petersen, T.E.; Randaccio, L. Structural Basis for Mammalian Vitamin B12 Transport by Transcobalamin. *Proc. Natl. Acad. Sci. U. S. A.* **2006**, *103*, 4386–4391, doi:10.1073/pnas.0509099103.

24. Bargelloni, L.; Babbucci, M.; Ferraresso, S.; Papetti, C.; Vitulo, N.; Carraro, R.; Pauletto, M.; Santovito, G.; Lucassen, M.; Mark, F.C.; et al. Draft Genome Assembly and Transcriptome Data of the Icefish Chionodraco Myersi Reveal the Key Role of Mitochondria for a Life without Hemoglobin at Subzero Temperatures. *Commun. Biol.* **2019**, *2*, 1–11, doi:10.1038/s42003-019-0685-y.
